# Diversity and Recognition Efficiency of T Cell Responses to Cancer

**DOI:** 10.1371/journal.pmed.0010028

**Published:** 2004-11-30

**Authors:** Tor B Stuge, Susan P Holmes, Sahdev Saharan, Andrea Tuettenberg, Mario Roederer, Jeffrey S Weber, Peter P Lee

**Affiliations:** 1Department of Medicine, Division of Hematology, Stanford UniversityStanford, CaliforniaUnited States of America; **2**Department of Statistics, Stanford UniversityStanford, CaliforniaUnited States of America; **3**Department of Dermatology, J. Gutenberg-UniversityMainzGermany; **4**ImmunoTechnology Section, Vaccine Research Center, National Institute of Allergy and Infectious Diseases, National Institutes of HealthBethesda, MarylandUnited States of America; **5**Norris Cancer Center, University of Southern CaliforniaLos Angeles, CaliforniaUnited States of America; National Jewish Medical and Research Center/Howard Hughes Medical InstituteUnited States of America

## Abstract

**Background:**

Melanoma patients vaccinated with tumor-associated antigens frequently develop measurable peptide-specific CD8+ T cell responses; however, such responses often do not confer clinical benefit. Understanding why vaccine-elicited responses are beneficial in some patients but not in others will be important to improve targeted cancer immunotherapies.

**Methods and Findings:**

We analyzed peptide-specific CD8+ T cell responses in detail, by generating and characterizing over 200 cytotoxic T lymphocyte clones derived from T cell responses to heteroclitic peptide vaccination, and compared these responses to endogenous anti-tumor T cell responses elicited naturally (a heteroclitic peptide is a modification of a native peptide sequence involving substitution of an amino acid at an anchor residue to enhance the immunogenicity of the peptide). We found that vaccine-elicited T cells are diverse in T cell receptor variable chain beta expression and exhibit a different recognition profile for heteroclitic versus native peptide. In particular, vaccine-elicited T cells respond to native peptide with predominantly low recognition efficiency—a measure of the sensitivity of a T cell to different cognate peptide concentrations for stimulation—and, as a result, are inefficient in tumor lysis. In contrast, endogenous tumor-associated-antigen-specific T cells show a predominantly high recognition efficiency for native peptide and efficiently lyse tumor targets.

**Conclusions:**

These results suggest that factors that shape the peptide-specific T cell repertoire after vaccination may be different from those that affect the endogenous response. Furthermore, our findings suggest that current heteroclitic peptide vaccination protocols drive expansion of peptide-specific T cells with a diverse range of recognition efficiencies, a significant proportion of which are unable to respond to melanoma cells. Therefore, it is critical that the recognition efficiency of vaccine-elicited T cells be measured, with the goal of advancing those modalities that elicit T cells with the greatest potential of tumor reactivity.

## Abstract 2

State-of-the art analysis of patients' response to melanoma vaccines yields lessons about cancer vaccines and rationale vaccine design in general

## Introduction

The immunotherapy of cancer holds promise in harnessing the host immune response to specifically target tumor cells without harming normal tissues. Strategies involve adoptive cellular therapy or active immune induction (commonly referred to as “cancer vaccination”). Cancer vaccines may consist of whole tumor cells or tumor lysates, but identification of tumor-associated antigens (TAAs) over the past decade has made possible the use of specific proteins or peptides as cancer vaccines. The anti-tumor potential of TAA-specific CD8+ T cells has been illustrated by the demonstrated capacity of adoptive T cell therapy to reduce tumor size [1]. While endogenous anti-tumor CD8+ T cell responses may already exist in some cancer patients [2], vaccination with TAA-derived peptides, and in particular heteroclitic peptide analogs, increases the frequency of TAA-specific T cell responses to detectable levels in many patients [3,4,5,6,7,8,9]. Heteroclitic peptide analogs are created by substitutions at anchor residues resulting in increased association of peptide with the major histocompatibility complex (MHC) [10]. Consequently, heteroclitic peptide analogs are predicted to be more immunogenic than their native counterparts because of more stable binding at the surface of antigen-presenting cells (APCs). Indeed, T cells capable of tumor lysis have been isolated from patients vaccinated with heteroclitic peptide [8,11,12,13]. However, the presence of TAA-specific T cells elicited by vaccination often does not correlate with clinical responses [3,14,15,16,17].

Various reasons for the paradoxical coexistence of cancer cells and TAA-specific T cells within patients have been proposed [18,19]. One possibility is that elicited TAA-specific T cells are not optimally functional in vivo [2,18]. Another possibility is that T cells inefficient in tumor recognition or lysis are induced by vaccination [20]. It is becoming recognized that antigen-specific T cells may have substantially different requirements for cognate peptide (the peptide that is recognizable to a specific T cell clone) for efficient target lysis [20,21,22,23]. “Recognition efficiency” (RE) (also known as “functional avidity”) is a measure of the sensitivity of a T cell to different peptide concentrations for stimulation [24,25,26]. We hypothesized that high antigen densities on APCs resulting from vaccination with heteroclitic peptide may paradoxically drive T cells of predominantly low RE, which are not efficiently activated by the endogenous expression levels of native peptides on tumor cells. Consequently, such T cells would be ineffective in tumor cell destruction. Support for this notion is emerging: T cells with low RE are predominantly expanded in vitro with high peptide concentration [22]. Moreover, in vitro stimulation of T cells from healthy donors with heteroclitic peptides results in expansion of cells with a wide range of RE [23]. A similar phenomenon may occur in vivo, leading to TAA-specific T cells of low RE depending on the nature of antigen stimulation [20].

While isolated T cell clones with low RE have indeed been generated from melanoma patients following heteroclitic peptide vaccination, the proportion of vaccine-elicited T cell responses these cells represent in vivo is not clear. If predominantly high-RE, tumor-cytolytic T cells are generated, then a small fraction of low-RE T cells generated would be of little consequence. However, if predominantly low-RE T cells are generated, then this low proportion of high-RE T cells may be an important factor in the observed lack of clinical effectiveness of current cancer vaccination strategies. To address this important issue, we undertook a systematic examination of the complexity of T cell responses induced by heteroclitic peptide vaccination, and compared these responses to endogenous anti-tumor T cell responses which develop in some patients. Typically, responses to vaccination are examined following in vitro expansion from patient samples, which may alter the composition of cells and consequently not reveal the proportion of cells in vivo having sufficiently high RE to lyse tumor targets. Although staining with peptide–MHC tetramers provides a direct estimate for the number of TAA-specific T cells present in vivo, and intensity of tetramer staining has been employed as a parameter for isolation of high-RE, tumor-lytic T cells [27], staining intensity does not correlate well with RE or tumor-lytic potential [28,29], and cannot be considered a reliable indicator for the functional status of TAA-specific T cells.

To analyze and compare T cell responses in melanoma patients on a single-cell level, we generated and examined a large number of cytotoxic T lymphocyte (CTL) clones derived from post-vaccination or endogenous anti-tumor T cell responses. Each clone was analyzed for T cell receptor (TCR) variable chain beta (VB) expression, RE, and ability to lyse melanoma targets. Importantly, these clones were generated directly ex vivo through tetramer-guided sorting, which minimizes the selection bias that could be introduced by prior in vitro expansion. Therefore, data from these clones could be taken to estimate the complexity of the responses in vivo.

## Methods

### Patients and Samples

All patients had resected stage III or IV melanoma, as determined by the 1988 modified American Joint Commission on Cancer staging system. They were required to have a magnetic resonance imaging or computed tomographic scan of the head and computed tomographic scans of the chest, abdomen, and pelvis showing no indication of disease within 4 wk of therapy to verify that they were clinically free of melanoma. Eligibility criteria included age 18 y or older, creatinine of less than 180 μmol/l, bilirubin of less than 110 μmol/l, platelet count of 100 × 10^9^/l or more, hemoglobin of 90 g/l or more, and total white blood cell count of 3.0 × 10^9^/l or greater. Tests for human immunodeficiency virus, hepatitis C antibody (Ab), and hepatitis B surface antigen were required to be negative, and all patients were HLA-A2 antigen positive by a microcytotoxicity assay. All patients were required to comprehend and sign an informed consent form approved by the National Cancer Institute (NCI; Bethesda, Maryland, United States) and the Los Angeles County/University of Southern California Institutional Review Board. Analysis of the patient samples was approved by Stanford University's Institutional Review Board. Peripheral blood mononuclear cell (PBMC) samples were isolated from patients after vaccination with the heteroclitic peptides MART 26–35 (27L) (ELAGIGILTV) and gp100 209–217 (210M) (IMDQVPSFV) at the University of Southern California Norris Cancer Center (Los Angeles, California, United States). Clinical-grade peptides used were provided by the Cancer Therapy Evaluation Program of the NCI under an Investigational New Drug application BB 6123 held by the NCI. Immunizations (1 mg of each peptide emulsified with incomplete Freund's adjuvant) were administered every 2 wk for 8 wk, then every 4 wk for 12 wk, and then once 8 wk later. PBMC samples were collected 4 wk after the final immunization and stored at −130 °C. Samples were thawed the day before an experiment for overnight culture in CTL medium. The following morning, viable cells were isolated by ficoll density centrifugation, washed, and resuspended to the appropriate concentration in a solution of 90% Iscove's Modified Dulbecco's Medium (IMDM) and 10% fetal bovine serum (FBS).

### Flow Cytometry Analysis

For isolation and detection of peptide-specific T cells, patient PBMC samples were stained and analyzed by fluorescence-activated cell sorting (FACS) as previously described [2]. Briefly, cells were stained with anti-human CD8− fluorescein isothiocyanate (Caltag Laboratories, Burlingame, California, United States) and CD19-CyChrome (BD Biosciences, Palo Alto, California, United States) Abs, and HLA-A*0201/peptide tetramer–phycoerythrin (PE). The final staining dilution of each Ab was 1/200 and 1/80, respectively. Tetramer–PE was titrated for optimal staining, usually between 1 and 10 μg/ml. For TCR VB typing, cells were divided in seven aliquots and stained with CD8 PerCP-Cy5.5 (BD Biosciences), tetramer–PE, and a panel of two or three different anti-VB monoclonal Abs labeled with fluorescein isothiocyanate, allophycocyanin (APC), or both. Cells were incubated at room temperature for 30 min, washed, then analyzed using a two-laser, four-color FACSCalibur (Becton Dickinson, Franklin Lakes, New Jersey, United States) or sorted using a FACSVantage flow cytometer (Becton Dickinson). Lymphocytes were identified by forward and side scatter signals, then selected for CD8+ and tetramer positive. Up to one million events were acquired and analyzed using FlowJo (TreeStar, San Carlos, California, United States).

### CD107 Mobilization Assays

#### Target cells

The HLA-A*0201-positive melanoma lines Malme-3M and A375 and the T2 cell line were purchased from ATCC (Manassas, Virginia, United States) and maintained according to instructions provided by the ATCC. The HLA-A*0201-positive melanoma line mel526 was obtained from the Surgery Branch of the NCI. While Malme-3M and mel526 express both MART and gp100, A375 does not express MART or gp100 and served as a negative control. Expression (or lack thereof) of these antigens by each cell line was further confirmed by immunohistochemical staining. Cells were trypsinized using Trypsin/EDTA solution (GIBCO, San Diego, California, United States) before use. T2 cells were HLA-A2.1+ and were pulsed prior to assays with peptides indicated in the text.

#### Effector cells

Effector cells, which include clones, cell line, and PBMC samples, were frozen and analyzed in batches. The cells were thawed the day before an experiment for overnight culture in CTL medium. The following morning, viable cells were isolated by ficoll density centrifugation, washed, and resuspended to the appropriate concentration (usually 10^7^/ml) in CTL medium.

#### Experimental procedure

All assays were done at least twice, with duplicates for each condition. The effector to target (E:T) ratio used was generally 1:2, with 2 × 10^5^ for clones or 10^6^ for the cell line and patient PBMC samples. To each well, the following was added in order: 1 μl of 2 mM monensin (Sigma, St. Louis, Missouri, United States) in 100% EtOH, 100 μl of target cells, 100 μl of effector cells, and 1 μl of CD107-APC Abs. The cells were mixed well using a multichannel pippetor. The plate was centrifuged at 300*g* for 1 min to pellet cells, then placed into an incubator at 37 °C for 4 h. After the incubation, the plates were centrifuged to 500*g* to pellet cells, and the supernatant was removed. Cell–cell conjugates were disrupted by washing the cells with PBS supplemented with 0.02% azide and 0.5 mM EDTA, and mixed vigorously, then stained with additional Abs.

### Generation of CTL Clones

CD8+ T cell clones were derived by FACSorting individual tetramer-positive cells from PBMC samples prepared for flow cytometry as described above. CD8+ tetramer-positive T cells were sorted under sterile conditions into 96-well plates, one cell per well, using a FACS Vantage (Becton Dickinson). Wells contained 100 μl of CTL IMDM, with 10% FBS, 2% human AB sera, and penicillin, streptomycin, and L-glutamine, supplemented with 100 units/ml IL-2. Sorted cells were expanded in vitro using standard protocols. Briefly, irradiated feeder cells (JY cells and fresh PBMCs) were added to wells containing the sorted T cells, and the 96-well plates were incubated at 37 °C with 7% CO_2_ to allow for growth. Potential clones became visible around day 14 and were then transferred to 24-well plates containing 1 ml of CTL medium with 100 units/ml IL-2. Wells were selected based on cell confluency for expansion and further analysis. Clones confirmed to be tetramer-positive were expanded in T-25 flasks containing irradiated JY cells and fresh PBMCs in 25 ml of CTL medium containing PHA. IL-2 was added to a final concentration of 50 units/ml on day 1 and then every 2 d thereafter for 2 wk.

### Cytotoxic Assays

#### Target cells

Target cells were as described above under CD107 Mobilization Assays, and were labeled overnight with ^51^Chromium, washed, and resuspended to 10^5^ cells/ml. One hundred microliters of target cells were incubated with 100 μl CTL clones at 10:1 E:T ratio for 4 h. Percent specific release of ^51^Chromium from target cells was calculated from 40-μl cell-free supernatants.

#### Determination of RE

Chromium-labeled T2 targets were pulsed with a range of peptide concentrations, generally starting at 10^−7^ M and decreasing by log steps to 10^−13^ M. T cell clones were incubated with T2 targets at 10:1 E:T ratios for 4 h, then chromium release was measured and percentage cytotoxicity calculated by standard methods. Prior to each cytotoxicity assay, clones underwent ficoll-hypaque centrifugation to remove dead feeder cells and were determined to be greater than 80% CD8+ tetramer-positive T cells by FACS. The E:T ratio was based upon live T and target cells. For each T cell clone, percent cytotoxicity was plotted against peptide concentration. The peptide concentration at which the curve crossed 40% cytotoxicity was defined as the RE of that clone [30].

#### Microcytotoxic assay

Cells were isolated directly from PBMCs from patient 422 by FACS as described above. Cells were collected in microfuge tubes containing 1 ml of ice-cold 90% IMDM with 10% FBS. Collected cells were washed and resuspended to 83,300 cells/ml in 90% IMDM with 10% FBS. Targets were prepared as described above and resuspended to 8,300 cells/ml in 90% IMDM with 10% FBS. A total of 2,500 sorted cells (30 μl) and 250 target cells (30 μl) were transferred to a microcentrifuge tube (VWR International, West Chester, Pennsylvania, United States), centrifuged 1 min at 200*g,* and incubated 4 h at 37 °C. Percent specific release of ^51^Chromium was calculated from 40 μl of cell-free supernatant.

### TCR VB Spectratyping

RNA was extracted from clones and tetramer-positive cells using TRIzol (Invitrogen, Carlsbad, California, United States) and reverse-transcribed into cDNA using SuperScript II Reverse Transcriptase (Invitrogen). PCR was performed using 34 different 5′ primers that specifically amplify all functional TCR VB genes. Most of the 5′ primers used have been previously described [31]. These primers were used in combination with a common 3′ primer based in the beta chain constant region, BC63 (5′- GTGTGGCCTTTTGGGTGT-3′). As an internal control, PCR for a section of the beta chain constant region was performed in parallel with VB-specific PCRs using the following primers: UpBC (5′- CGCTGTGTTTGAGCCATC-3′) and LoBC (5′- TGCTCAGGCAGTATCTGGA-3′). All primer concentrations were 200 nM. PCR was performed using an iCycler iQ thermic cycler equipped with a real-time detection system (Bio-Rad, Hercules, California, United States) and a QuantiTect SYBR Green PCR kit (Qiagen, Valencia, California, United States). PCR reactions were performed as follows: 94 °C for 9 min, followed by 50 cycles of 94 °C for 30 s, 58 °C for 1 min, and 72 °C for 1 min, followed by 72 °C for 10 min. Specific amplification was determined relative to constant region control PCR. For spectratyping, PCRs were performed as described above with the following VB14- and VB17-specific 5′ primers: VB14m (5′- ACCCAAGATACCTCATCACAG-3′) and VB17 (5′- GACAGGACCCAGGGCAAG-3′), followed by a run-off PCR with downstream VB-specific primers: VB14 (5′- GGGCTTAAGGCAGATCTACT-3′) and VB17m (5′- TTTCAGAAAGGAGATATAGCT-3′), and FAM6-labeled BC63 3′ primer. Run-off PCR was performed as described above except that only five cycles of PCR were run with the 55 °C annealing temperature and QuantiTect Probe PCR kit (Qiagen). Labeled PCR fragments were run on an ABI Prism 377 DNA Sequencer (Applied Biosystems, Foster City, California, United States) and analyzed using GeneScan software (Applied Biosystems).

### Statistical Analysis

A standard software package (SigmaPlot 5.0, Systat Software, Richmond, California, United States) was used to provide descriptive statistical plots. Barcharts were provided with standard errors on them. Linear plots were provided with standard errors computed at each point. A linear regression (using least squares) of percent specific lysis on recognition efficiency is shown in Figure 5A and 5B.

**Figure 4:**
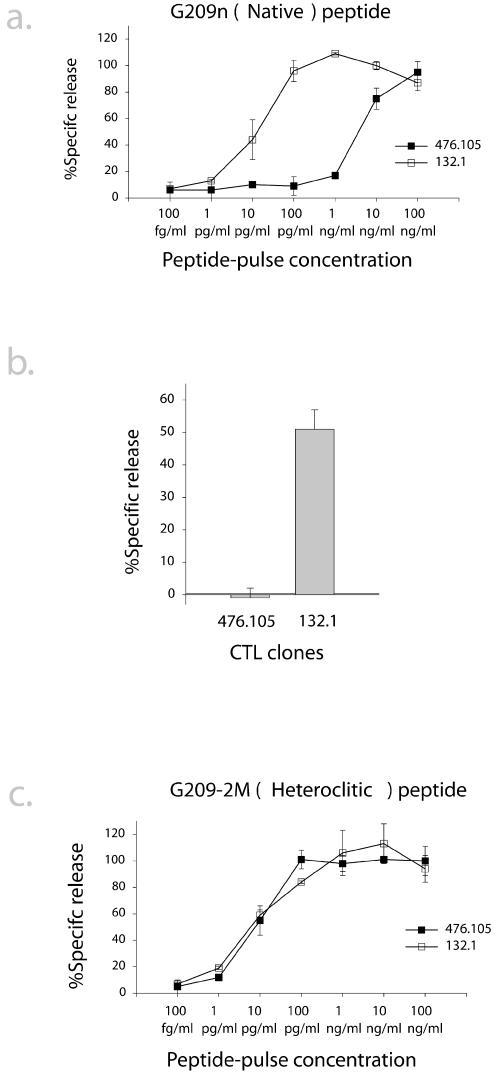
High RE Recognition of Native G209n but Not G209–2M Peptide Correlates with Efficiency in Tumor Cell Lysis CTL clones 476.105 and 132.1 were assayed for lysis of T2 cells pulsed with 10-fold dilutions of (A) native or (C) heteroclitc peptide at concentrations ranging from 100 fg/ml to 100 ng/ml. (B) Lysis of Malme-3M melanoma cells by 476.105 and 132.1 CTLs. All assays were performed in triplicate, and each clone was assayed twice. Error bars reflect variation between two separate assays.

**Figure 5:**
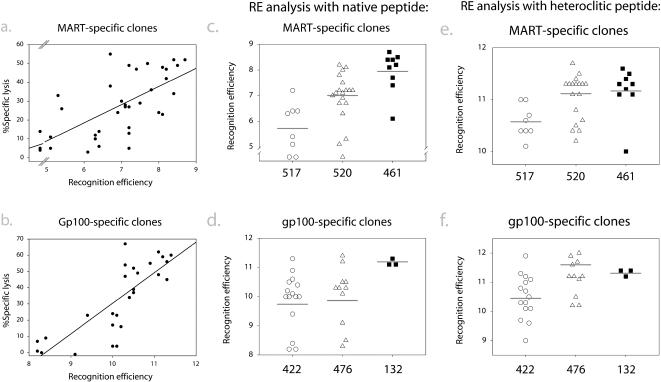
Endogenous T-Cell Responses Have Higher RE Than Vaccine-Elicited Responses CTL clones representing different tetramer-positive populations in each patient expressing different VB were assayed for lysis of T2 cells pulsed with various dilutions of G209n, G209–2M, M27, or M26 peptides in ^51^Chromium release cytotoxicity assays as described in Figure 4 legend. A RE score was attributed to each clone equal to the negative log_10_ of the peptide concentration that resulted in 40% lysis of peptide-pulsed T2 cells. (A and B) RE scores for both (A) MART-specific and (B) gp100-specific clones from all patients were correlated with efficiency in lysing melanoma cells. Correlation coefficients were 0.66 for MART-specific clones and 0.81 for gp100-specific clones. (C–F) Comparison of RE scores for endogenous (patients 461 and 132) and vaccine-induced (patients 517, 520, 422 and 476) responses. (C and D) RE analysis with native peptides (C) M27 and (D) G209n. Mean RE (weighted) for each response is indicated with horizontal bars. Weighted means were based on all clones, not only those assayed, and were estimated by summing the RE of each analyzed clone multiplied by the number of total clones expressing the same VB, in each patient. Weighted means were as follows: patient 517, 5.7; patient 520, 7.0; patient 461, 7.9; patient 422, 9.7; patient 476, 9.9; and patient 132, 11.2. One-tailed T-tests demonstrated that endogenous responses had significantly higher RE than vaccine-induced responses: patient 461 versus patient 517, *p* = 1.8 × 10^−5^; patient 461 versus patient 520, *p* = 1.1 × 10^−3^; patient 132 versus patient 422, *p* = 6 × 10^−6^; and patient 132 versus patient 476, *p* = 4.3 × 10^−4^. (E and F) RE analysis with heteroclitic peptides (E) M26 and (F) G209–2M. Weighted means were as follows: patient 517, 10.6; patient 520, 11.1; patient 461, 11.2; patient 422, 10.5; patient 476, 11.6; and patient 132, 11.3.

## Results

### T Cell Responses to TAAs in Patients with Melanoma

**Figure 1:**
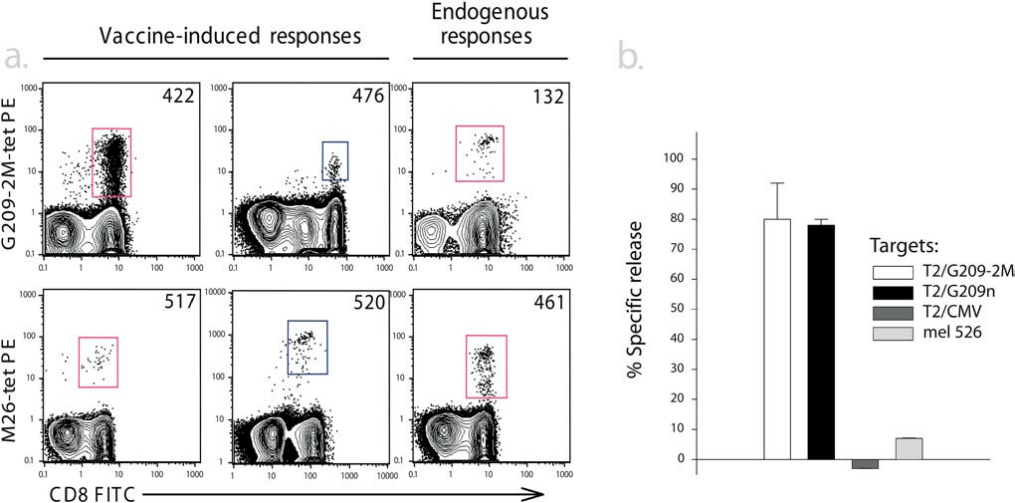
Melanoma Patient Samples Selected for Analysis of RE for Melanoma Cells (A) Six patients with T cell responses reactive with for M26 or G209–2M tetramers were selected for analysis. PBMCs from each patient were stained with PE-conjugated peptide–MHC tetramers, G209–2M-tet PE or M26-tet PE, and co-stained with anti-CD8 fluorescein isothiocyanate and anti-CD14, -CD19, and -CD4 Cy5PE. The plots shown are gated for CD8+, CD14−, CD19−, and CD4− cells. Tetramer-positive cells are boxed and estimated for percent of total CD8+ cells: patient 422, 2.5%; patient 476, 0.31%; patient 132, 0.22%; patient 517, 0.23%; patient 520, 0.12%, and patient 461, 0.50%. (B) Microcytotoxicity ^51^Chromium release assay with tetramer-positive cells isolated by FACS from the CD8+ PBMC population from patient 422. Isolated cells were assayed for lysis of T2 cells treated with relevant or irrelevant peptide, or mel526 melanoma cells. Sorted cells were combined with 250 target cells at 13:1 E:T ratios for 4 h, and supernatants were assayed for percent specific release of radiolabel.

To address the complexity of T cell responses against melanoma in vivo, patients with vaccine-induced or endogenous TAA-specific responses were selected. In recent cancer vaccine trials [3,4,5], many melanoma patients who received heteroclitic peptide vaccines gp100 209–217 (210M) (IMDQVPSFV; G209–2M) and MART 26–35 (27L) (ELAGIGILTV; M26) had measurable CD8+ peptide-specific T cell responses in PBMCs detected by peptide–MHC tetramer staining. In addition, TAA-specific T cell responses could be detected in some patients without vaccination, suggesting the existence of an endogenous anti-tumor T cell response in these patients. For the current study, we selected samples from six melanoma patients from these trials—four with vaccine-elicited responses (patients 422, 476, 517, and 520) and two with endogenous T cell responses (patients 132 and 461)—for detailed analyses of TCR VB usage, RE for the target peptide, and tumor cytotoxicity. The samples from these six patient had peptide-specific T cell populations detectable with G209–2M-tetramers (patients 422, 476, and 132) or M26-tetramers (patients 517, 520, and 461) ranging from 0.1% to 2.5% of total CD8+ T cells (Figure 1A).

### Vaccine-Elicited T Cells Are Functional Directly Ex Vivo but of Variable Tumor Reactivity

Patient 422 had the largest detectable TAA-specific CD8+ T cell response (2.5% G209–2M-tetramer-positive) and thus sufficient numbers for examination of lytic function immediately following isolation. To test whether peptide-vaccine-induced T cell responses were functionally active directly ex vivo, T cells isolated by G209–2M-tetramer-guided cell sorting from patient 422 were tested for lysis of peptide-pulsed and melanoma target cells in microcytotoxic assays (Figure 1B). The directly isolated tetramer-positive T cells from this patient specifically lysed T2 cells pulsed with high concentrations (1 μg/ml) of G209–2M and native (G209n) peptides, but not with T2 cells pulsed with a cytomegalovirus-derived, HLA-A*0201-restricted peptide (NLVPMVATV) or melanoma targets. This suggests that while a significant portion of the vaccine-elicited T cells from patient 422 may be functional in vivo, they did not have significant tumor lysis activity.

To assess the functional status of the smaller TAA-specific CD8+ T cell responses in the other five patients—which were too small for direct cytotoxicity assays after sorting—we utilized a novel FACS assay for degranulation based on CD107 mobilization [24]. All six TAA-specific populations exhibited robust functional responses ex vivo, as measured by percentage of G2090–2M- and M26-tetramer-positive cells that mobilized CD107 and/or downregulated the CD3 complex upon incubation with T2 cells pulsed with cognate peptides (Table 1; 86%-99.6%). In response to melanoma targets mel526 and Malme-3M, which both express gp100 and MART-1 and are HLA-A*0201 positive, the two endogenous TAA-specific responses (samples from patients 132 and 461) also exhibited robust functional responses directly ex vivo (Table 1; 36.8%–87%), and these responses were specific as they had little response to A375, a HLA-A*0201-positive melanoma cell that does not express gp100 or MART-1 and served as a negative control for antigen-specific killing (Table 1; 2.7% and 3%). In contrast, the vaccine-elicited responses exhibited much lower reactivity to mel526 and Malme-3M (Table 1; 23.8%–32.5%). These data demonstrate that all six TAA-specific CD8+ T cell responses were functional ex vivo, but there were significant differences in reactivity to melanoma targets between endogenous and vaccine-elicited responses.

**Table 1:**
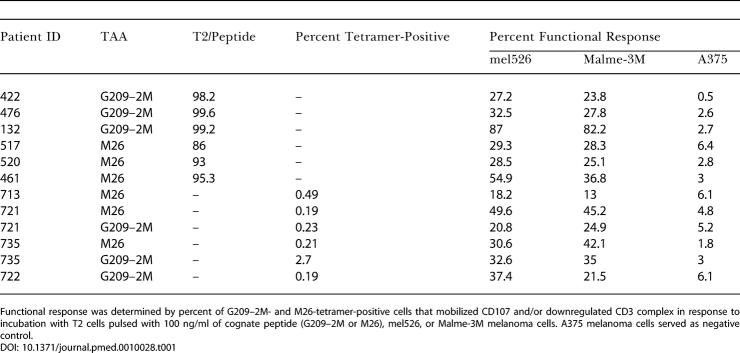
Functional Status of TAA-Specific T Cell Response

Functional response was determined by percent of G209–2M- and M26-tetramer-positive cells that mobilized CD107 and/or downregulated CD3 complex in response to incubation with T2 cells pulsed with 100 ng/ml of cognate peptide (G209–2M or M26), mel526, or Malme-3M melanoma cells. A375 melanoma cells served as negative control

To substantiate the generality of these findings, we analyzed four additional patients with vaccine-elicited responses. One subject responded to G209–2M only (patient 722), one to M26 only (patient 713), and two to both G209–2M and M26 (patients 721 and 735). Similar to the first four vaccine-elicited patients, these four additional patients (six TAA-specific responses in total) exhibited variable reactivity to melanoma targets, ranging from 13% to 49.6% (Table 1).

### Vaccine-Elicited T Cells Have Varied Capacity to Lyse Melanoma Targets

To confirm and further investigate the differences in tumor reactivity between endogenous and vaccine-elicited responses, we reasoned that analysis of a set of clonal CTL lines that represented the tetramer-positive population would provide an accurate estimate of the complexity of the TAA-specific T cell response in each patient. A large number of clonal CTL lines (more than 200) were generated by FACS of individual G209–2M- and M26-tetramer-positive cells directly from PBMC samples (Table 2). Up to 85% of sorted cells expanded in various sorts (data not shown). Randomly selected expanding clones and the tetramer-positive population from which they were derived were examined for TCR VB expression using TCR VB-specific monoclonal Abs and VB-specific primers in PCR. Diverse TAA-specific T cell responses were found in the four vaccinated patients, with multiple T cells expressing different TCR VB, while the two endogenous responses were less diverse. All but one clone derived from patient 132 expressed VB17, while two dominating T cell populations in patient 476 expressed VB14 and VB17 (Table 2). The clonality of the dominant populations in these patients was evaluated by PCR fragment length analysis (Table 3). Identical length fragments were demonstrated in the four selected clones from 476 BV14+ and 476 BV17+ populations. Identical length fragments were also demonstrated in all BV17+ clones from patient 132. Furthermore, analysis of sorted tetramer-positive cells from patient 476 demonstrated single fragment sizes for BV14 and BV17, which were identical to the fragment sizes generated from the selected clones, arguing for clonality of these dominant populations (Table 3).

**Table 2:**
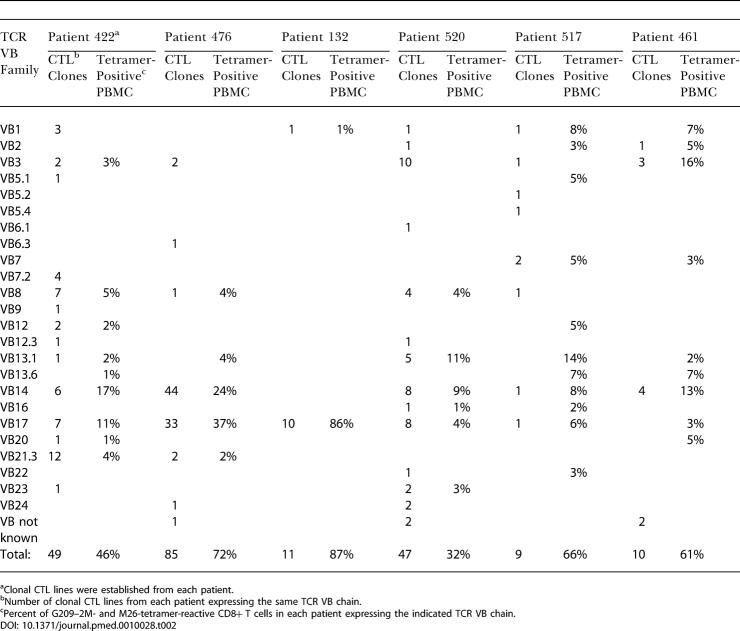
CTL Clones Established from Each Patient Represent a Random Selection from the Tetramer-Reactive CD8+ Parent Population

^a^Clonal CTL lines were established from each patient

^b^Number of clonal CTL lines from each patient expressing the same TCR VB chain

^c^Percent of G209–2M- and M26-tetramer-reactive CD8+ T cells in each patient expressing the indicated TCR VB chain

**Table 3:**
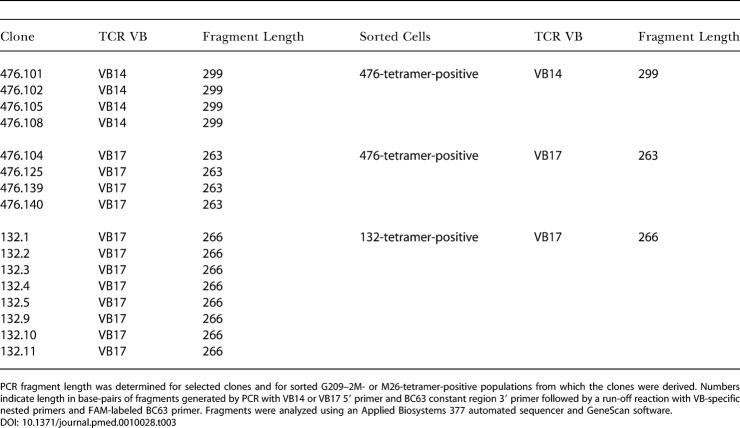
PCR-Generated Fragment Length Analysis

PCR fragment length was determined for selected clones and for sorted G209–2M- or M26-tetramer-positive populations from which the clones were derived. Numbers indicate length in base-pairs of fragments generated by PCR with VB14 or VB17 5′ primer and BC63 constant region 3′ primer followed by a run-off reaction with VB-specific nested primers and FAM-labeled BC63 primer. Fragments were analyzed using an Applied Biosystems 377 automated sequencer and GeneScan software

**Table 4:**
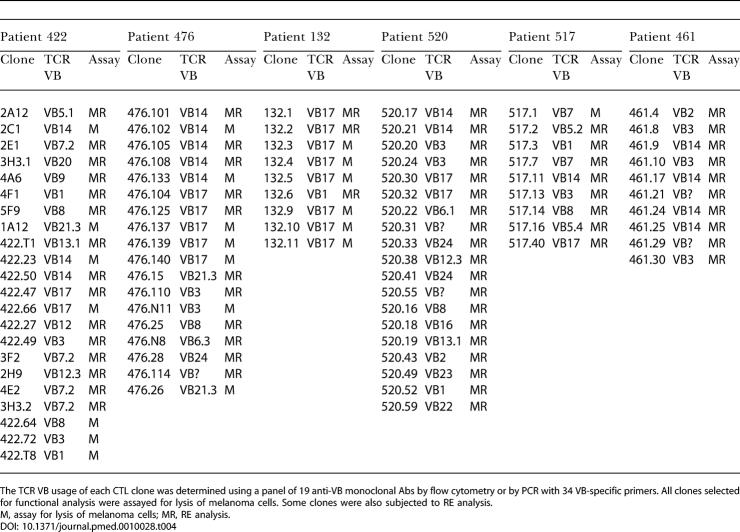
CTL Clones from Each Patient Selected for Functional Analysis

The TCR VB usage of each CTL clone was determined using a panel of 19 anti-VB monoclonal Abs by flow cytometry or by PCR with 34 VB-specific primers. All clones selected for functional analysis were assayed for lysis of melanoma cells. Some clones were also subjected to RE analysis

M, assay for lysis of melanoma cells; MR, RE analysis

Peptide specificity and CD8 expression of each clone was confirmed by staining with G209–2M- and M26-tetramers and anti-CD8 monoclonal Ab (data not shown). To obtain an accurate reflection of the total T cell population detected with tetramer in each patient, we decided to rigorously examine at least one representative clone for each subpopulation expressing a different TCR VB (Table 4). Multiple clones were analyzed to determine dominating populations. From patients 132, 517, and 461, for which fewer clones were generated, all clones were included in the analyses (Table 4).

**Figure 2:**
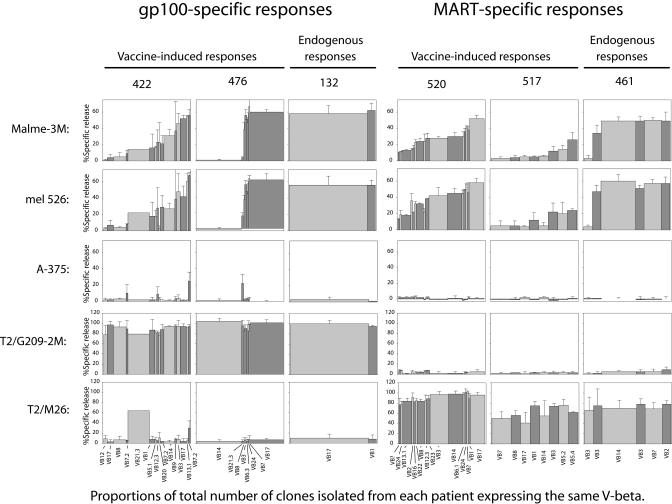
Endogenous T Cell Responses Are More Efficient in Melanoma Lysis Than Vaccine-Elicited Responses Cells from 87 clonal CTL lines were assayed for lysis of melanoma cells mel526, Malme-3M, and A375 in ^51^Chromium release cytotoxicity assays. Mel526 and Malme-3M are HLA-A2.1+ and express both gp100 and MART-1. A375 cells are HLA-A2.1+ but do not express either gp100 or MART-1 and served as a negative control. T2 cells treated with 1 μg/ml G209–2M or M26 peptides served as controls for antigen-specific lysis. The CTL clones assayed were selected to represent different tetramer-positive subsets expressing different VB. Dominating tetramer-positive populations in each patient were represented with two or more clones. Each CTL clone was assayed in triplicate wells, and the data displayed are averages of two different experiments. Clones from the same patient expressing similar VB while exhibiting different lysis potential were viewed as separate subsets. Each assay was performed at 10:1 E:T ratio as detailed in Methods. The height of each bar represents percent specific lysis, while the width represents the relative size of the tetramer-positive subpopulations (defined by VB expression) in each patient. Population size was defined as the percent of clones from each patient expressing the same VB. Error bars show standard deviation between two experiments within each clone and/or between different clones where more than one clone was analyzed.

**Figure 3:**
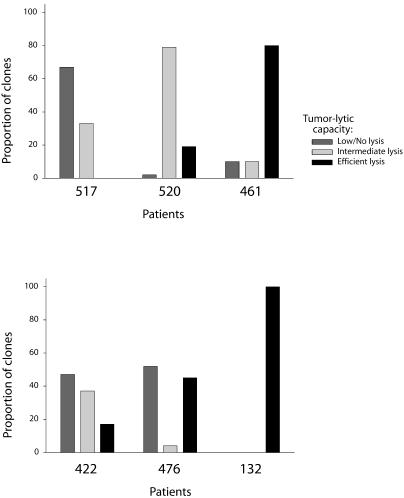
Most CTL Clones Isolated from Endogenous Responses Are Efficient in Tumor Cell Lysis CTL clones derived from each patient were classified as “efficient” (greater than 40%), “intermediate” (between 10% and 40%), or “low/no” (less than 10%) in lysis of melanoma cells based on data displayed in Figure 2. Each bar represents the portion of total clones from each patient with “efficient,” “intermediate,” or “low/no” melanoma lysis potential.

To determine the effectiveness of tumor lysis by the different TAA-specific T cell clones that were propagated, clones were analyzed for their ability to lyse melanoma cell lines mel526 and Malme-3M. A375 cells served as a control for antigen-specific killing. In addition, each CTL clone was examined for antigen-specific lysis of T2 cells pulsed with high levels (1μg/ml) of G209–2M or M26 peptides. “Efficient lysis” in these experiments was defined as 40% or greater specific release of radiolabel from the target cells; 10% or less specific release was categorized as “low or no lysis,” and 10% to 40% was termed “intermediate lysis.” All but two of the CTL clones elicited from endogenous anti-tumor responses (from patients 132 and 461) exhibited “efficient lysis” of both the mel526 and Malme-3M melanoma cell lines (Figure 2). In contrast, only a few clones from the vaccine-elicited responses (from patients 422, 476, 520, and 517) efficiently lysed melanoma cells. The majority of clones examined from these vaccine-elicited responses either failed to lyse melanoma targets altogether or lysed them with intermediate efficiency (Figure 2). This lack of efficiency in melanoma cell lysis was not due to cellular dysfunction, since each clone efficiently lysed T2 cells pulsed with high levels of relevant, but not irrelevant, peptide (Figure 2). Overall, the majority of clones derived from endogenous anti-tumor responses (patients 132 and 461) lysed both mel526 and Malme-3M melanoma target cells more efficiently than clones from vaccine-elicited responses (patients 422, 476, 520, and 517) (Figure 3). These findings suggest that TAA-specific T cells elicited by heteroclitic peptide vaccination have different tumor-cytolytic potentials from those which develop endogenously to cancer.

### RE for Native and Heteroclitic Peptides of T Cells from Endogenous or Vaccine-Elicited Responses

We hypothesized that CTL clones that did not efficiently lyse melanoma targets may be incapable of recognizing the relatively low surface densities of native peptide present on tumor cells. CTL clones selected for analysis of tumor lysis were also assessed for RE for the native and heteroclitic peptides via a ten-log range of dilutions. This is illustrated with clones 132.1 and 476.105 (Figure 4A). There were considerable differences in killing of peptide-pulsed T2 cells by these two clones. The differences in RE for G209n native peptide displayed by the two clones highlighted in Figure 3A correlated with their ability to lyse melanoma cells: the high-RE clone 132.1 efficiently lysed melanoma targets, whereas the low-RE clone 476.105 did not (Figure 4B). In contrast to the differences in RE for G209n peptide, similar assays revealed little difference in RE of the two clones for G209–2M heteroclitic peptide (Figure 4C), suggesting that these clones recognize the native and heteroclitic peptides differently, and that RE for the native, but not heteroclitic, peptide correlates with tumor-lytic potential.

Similar RE assays were performed for the remaining clones from each patient selected for analysis. In order to compare REs of various CTL lines, each clone was assigned an RE score expressed as the negative log_10_ value of the peptide concentration required for 40% specific lysis at an E:T ratio of 10:1. For clones 132.1 and 476.105, these scores were 11.1 and 8.3 for assays with G209n peptide (Figure 4A), and 11.2 and 11.2 for assays with G209–2M heteroclitic peptide (Figure 4C), respectively. We compiled the data on clones from all patients, which showed a correlation between tumor-lytic potential and RE for native peptide (Figure 5A and 5B). Overall, clones generated from endogenous anti-tumor responses had higher RE for the native peptide than clones generated from post-vaccine responses (Figure 5C and 5D). We estimated the composite RE of the overall TAA-specific response (composed of a heterogeneous population of T cells) in vivo by summing the RE of each clone multiplied by its representation in the original mixture (the representation was estimated based on the proportion of TAA-specific cells expressing the same VB as the clone). These composite RE values are represented in Figure 5 as horizontal bars for each response. Clearly, the endogenous responses (patients 461 and 132) had a higher overall, and more homogeneous, RE for the native peptide than the vaccine-elicited responses (patients 422, 476, 517, and 520) (Figure 5C and 5D). Importantly, the vaccine-elicited clones also exhibited wide variations in RE even for the heteroclitic peptide, compared to the endogenous responses (Figure 5E and 5F). This suggests that the variation in RE for native peptides, and hence ability to lyse tumor cells, for vaccine-elicited responses is not merely a reflection of differential recognition of native and heteroclitic peptides by many clones. Rather, variations in RE may be a function of the manner in which these cells were elicited in vivo via vaccination.

## Discussion

To achieve maximal clinical responses, the majority of T cells elicited by vaccination in cancer patients should be capable of responding to tumor targets. We have undertaken the most detailed analysis to date, on a single-cell level, of T cell responses elicited by cancer vaccination and have compared these with endogenous anti-tumor responses. To evaluate the full spectrum of T cells elicited in each patient by vaccination, we utilized tetramers made with the vaccine peptides (heteroclitic M26 and G209–2M) to isolate such cells. CTL clones were selected directly from patient PBMC samples without enrichment in culture to closely reflect the composition of the antigen-specific T cell response in vivo at the time of isolation.

Our data revealed that T cell populations induced by vaccination were significantly different from endogenous responses: while some CTLs elicited by vaccination could kill melanoma targets, most were inefficient in tumor cell lysis. In contrast, nearly all clones from endogenous responses were efficient at melanoma cell lysis. This difference was related to RE for the native peptide. Clones that did not lyse tumor cells required up to 10^3^-fold higher concentration of peptide for similar levels of lysis of targets compared to T cell clones that were tumor-lytic. Side-by-side comparison of endogenous responses and vaccine-induced responses suggests that low RE TAA-specific T cell responses may be preferentially driven by heteroclitic peptide vaccination. Thus, high doses of peptide and/or the higher levels of expression of heteroclitic peptide on APCs may induce and actively propagate predominantly T cells with RE too low for recognition of physiological levels of the native peptide present on tumor targets. These data suggest an inverse relationship between antigen density and the RE of T cells elicited. This would be an important consideration in design of future vaccine strategies.

Differential recognition of native and heteroclitic peptides by many T cells may also account for the induction of non-tumor-lytic clones by heteroclitic peptide vaccines, which has been suggested previously [23,32]. However, our data suggest that epitope density may be the dominant driving factor for RE in vivo. In all of the vaccine-elicited T cell responses, many of the T cells generated were either of low or intermediate RE not only for the native peptide, but also for the heteroclitic peptide, and exhibited no or intermediate lysis of tumor targets. In contrast, nearly all of the clones generated from the endogenous responses were of high RE. This suggests that the high dosage of peptides administered in vaccinations and the increased binding capacity of heteroclitic peptides to MHC molecules—the very quality that provides them with increased immunogenicity—drive the induction of many T cells with low RE for both heteroclitic and native peptides.

Another implication of this study is that the number of cells measured by current methods, including ELISPOT or staining with MHC tetramers, may not correlate directly with the RE or tumor reactivity of T cell responses to vaccination. For example, of the nine clones analyzed from patient 517, none were efficient in tumor cell lysis, yet these cells were detectable by MHC tetramer staining. T cells with low RE for native TAA do not efficiently lyse tumor, and therefore are unlikely to have an impact on clinical outcome. Furthermore, it may be possible that low-RE TAA-specific T cells may interfere with elicitation of high-RE T cells, either by direct competition for antigen on APC surface [33,34] or down-modulation of peptide–MHC complexes.

Our data support the notion that not only quantity, but quality, of the T cell response elicited by vaccination may be important for clinical efficacy. There are a number of strategies to increase the magnitude of T cell responses to peptide vaccines. These include using various adjuvants, such as incomplete Freund's adjuvant and immunomodulatory agents, such as IL-12 [4], GM-CSF [5], anti-CTLA-4 Abs [35], or heat shock proteins [36]. Thus far, none of these approaches have produced improved clinical outcomes. Our data suggest that in addition to driving higher numbers of vaccine-elicited T cells, strategies to modulate the relative RE of T cell responses are also needed. While the selective activation of high- versus low-RE T cells is relatively easy to manipulate in vitro via stimulation with limiting amounts of peptides, this may be more difficult to control in vivo. It is important to bear in mind that signals needed to drive a de novo naïve T cell response may be different from those required to drive further expansion of an activated T cell population [37]. Thus, a complete vaccination strategy may involve an initial induction phase, followed by progressive shaping of the response to higher RE. Although heteroclitic peptide vaccination may drive T cells of mixed high and low RE, such a strong stimulus may be needed to induce an initial de novo T cell response. Studies in mice suggest that once activated, effector CD8+ T cells may have an increase in RE of up to 70-fold compared to naïve cells [38,39,40]. Thus, naïve TAA-specific T cells, with inadequate RE to become activated by low densities of native peptides present on tumor cells, may become efficient in tumor lysis upon vaccination with heteroclitic peptide. This notion has support from studies in tolerized mice: vaccination with a heteroclitic peptide analog recruited T cells, which were responsive to secondary stimulation with native peptide [41,42]. Therefore, optimized use of heteroclitic peptide to induce an initial peptide-specific T cell response, followed by selective expansion of the highest RE tumor-lytic T cells may be needed for an effective strategy with clear clinical application.

In summary, we have demonstrated that vaccination with heteroclitic peptide at high concentrations may drive T cell responses of variable tumor-cytolytic potential in cancer patients—and that the ability to lyse tumor cells correlates with the T cell's RE for native peptides. This represents an important—but not sole—factor in explaining the lack of correlation between immunological and clinical responses after vaccination for cancer. Importantly, the situation is different in endogenous responses, in which cells are predominantly of high RE. This suggests that the manner in which T cells are elicited in vivo are different in these two settings and may underlie their differences in biology.

**Patient Summary**

**Why Was This Study Done?**

Our immune system protects us against infectious diseases. It can also recognize and destroy early cancer cells before they form tumors. Researchers have been trying to find a way to boost the anti-cancer function of the immune system so that it can kill even established tumors. This is the idea behind developing vaccines for treating cancer—the vaccine alerts and boosts the patient's immune system and so helps to fight the cancer. The idea of enlisting the immune system against cancer has been around for a long time. There have been some spectacular successes, but it has proven difficult to find vaccines that work in more than just a few patients. And we don't yet understand why vaccines seem to work in some patients but not in others.

**What Did the Researchers Do?**

Peter Lee and colleagues are trying to find out why some patients respond to vaccines and others don't by looking at the immune response in vaccinated patients. In this study, using state-of-the art technology, they examined patients who received different vaccines against the skin cancer melanoma. They concentrated on the so-called killer T cells (cytotoxic T cells), which directly attack and kill tumor cells, and analyzed them in great detail.

**What Did They Find?**

Most cytotoxic T cells produced by patients after vaccination—including vaccination with so-called heteroclitic peptides that had been specifically designed to provoke a very strong immune response—did not kill tumor cells very well, but a few of them did. These results provide some explanation as to why cancer vaccines haven't been as successful as many had hoped, but also suggest that if it were possible to get more of the potent T cells or to expand the ones that are already produced with the current vaccines, there would be a stronger anti-tumor response.

**What Next?**

How to get effective cancer vaccines remains an open question. But at least technologies such as those used in this study now exist that allow researchers to analyze how the immune systems of different patients react to vaccination and hence can guide the development of better vaccines.

**Additional Information.**

The Cancer Research Institute: http://www.cancerresearch.org/

US Food and Drug Administration page on cancer vaccines: http://www.cancerresearch.org/

University of Michigan page on cancer vaccines: http://www.cancer.med.umich.edu/learn/cancervaccines.htm

## Abbreviations

Ab: antibody
APC: antigen-presenting cell
CTL: cytotoxic T lymphocyte
E:T: effector to target
FACS: fluorescence-activated cell sorting
FBS: fetal bovine serum
IMDM: Iscove's Modified Dulbecco's Medium
MHC: major histocompatibility complex
NCI: National Cancer Institute
PE: phycoerythrin
PBMC: peripheral blood mononuclear cell
RE: recognition efficiency
TAA: tumor-associated antigen
TCR: T cell receptor
VB: variable chain beta
